# Dysregulation of Plasma miR-146a and miR-155 Expression Profile in Mycosis Fungoides Is Associated with rs2910164 and rs767649 Polymorphisms

**DOI:** 10.3390/ijms24010271

**Published:** 2022-12-23

**Authors:** Chrysostomos Avgeros, Aikaterini Patsatsi, Dimitrios Dimitriadis, Andigoni Malousi, Triantafyllia Koletsa, Despoina Papathemeli, Antonia Syrnioti, Paraskevi Avgerou, Elizabeth Lazaridou, Georgios Tzimagiorgis, Elisavet Georgiou

**Affiliations:** 1 Laboratory of Biological Chemistry, School of Medicine, Faculty of Health Sciences, Aristotle University of Thessaloniki, 54124 Thessaloniki, Greece; 2 2nd Dermatology Department, School of Medicine, Faculty of Health Sciences, Aristotle University of Thessaloniki, “Papageorgiou” General Hospital, 56403 Thessaloniki, Greece; 3 Center for Interdisciplinary Research and Innovation (CIRI-AUTH), 57001 Thessaloniki, Greece; 4 School of Economics, Aristotle University of Thessaloniki, 54124 Thessaloniki, Greece; 5 Department of Pathology, School of Medicine, Faculty of Health Sciences, Aristotle University of Thessaloniki, 54124 Thessaloniki, Greece

**Keywords:** miRNAs, mycosis fungoides, single-nucleotide polymorphism, CTCL

## Abstract

Diagnosis of Mycosis Fungoides (MF) may be challenging, due to its polymorphic nature. The use of miRNAs as biomarkers to assist in diagnosis has been investigated, mainly in skin lesion biopsies. The purpose of this study is to evaluate the plasma levels of miR-146a and miR-155 in MF patients and to investigate their association with SNPs of their genes. Plasma miRNAs were quantified by RT-qPCR. Genomic DNA was used for SNPs’ genotyping by Sanger sequencing. Plasma levels of miR-146a and miR-155 were significantly higher in patients vs. controls, in early MF patients vs. controls, and in advanced vs. early MF patients. Both miRNAs’ levels were significantly higher in stage IIB vs. early-stage patients. miR-155 plasma levels were significantly higher in patients with skin tumors or erythroderma. CC genotype (rs2910164 C>G) was significantly more frequent in healthy controls and associated with lower MF risk and lower miR-146a levels. The AA genotype (rs767649 T>A) was significantly more frequent in patients and correlated with increased MF risk and increased miR-155 levels. The combination of GG+AA was only detected in patients and was correlated with higher MF susceptibility. Increased mir-146a and mir-155 plasma levels in MF is an important finding to establish putative noninvasive biomarkers. The presence of SNPs is closely associated with miRs’ expression, and possibly with disease susceptibility.

## 1. Introduction

Mycosis Fungoides (MF) is the most prevalent primary Cutaneous T Cell Lymphoma subtype, comprising 55–64% of cases [1]. The clinical spectrum of MF is considerably variable. Most patients have early-stage MF, displaying erythematous patches or infiltrated plaques. Nevertheless, a quarter of patients progress to advanced-stage with tumors, erythroderma, and systemic involvement [2,3,4,5]. Diagnosis is mostly based on specific histopathological and immunohistochemical findings, while clinicopathological correlation is essential. However, due to its polymorphic nature, especially at early stages, repeated skin biopsies are often required to minimize the possibility of mis- or late diagnosis [6], while molecular analysis and the discovery of novel biomarkers may contribute to the accuracy of diagnosis.

MicroRNAs (miRNAs) are small (~18–25 nucleotides) non-coding RNA molecules that regulate genes’ expression [7]. Due to their distinct expression profile, miRNAs have been suggested as putative biomarkers in many malignancies, including MF [8,9]. Two of the most well-studied and highlighted miRNAs in MF are the inflammation-related miR-146a and miR-155 [10,11,12], which play a key role in the pathogenesis of the disease [13,14]. Circulating miRNAs display high stability and resistance to degradation, featuring as possible non-invasive biomarkers [15]. Nevertheless, there are only two studies evaluating miRNA plasma levels in MF, in a limited number of patients [16,17].

Single-nucleotide polymorphisms (SNPs) are common genetic variations that affect miRNAs’ expression. Two SNPs, rs2910164 (C>G) in pre-mir-146a and rs767649 (T>A) in a regulatory region of miR-155, have been associated with the expression of these miRNAs in benign and malignant diseases [18,19,20,21]. There are no studies in MF to date that associate miRNAs’ expression with SNPs in their genes.

In the present study, we evaluated plasma levels of miR-146a and miR-155 in MF patients to investigate their potential role as non-invasive prognostic and diagnostic biomarkers. Furthermore, we examined whether rs2910164 and rs767649 affect the miRNAs’ levels in plasma and susceptibility to MF.

## 2. Results

### 2.1. Demographic and Clinical Characteristics of Patients Recruited in This Study

A total of 41 patients were recruited (30 male, 11 female). The median age was 59 years. Among them, 11 had patch-stage disease, 21 plaque-stage, 6 tumor-stage and 3 were erythrodermic. According to EORTC staging, 32 (~78%) patients had early-stage MF (IA–IIA) and 9 (~22%) patients had advanced-stage MF (IIB-IV). Furthermore, 19 patients had not received any treatment prior to sample collection, while 22 had received skin-directed and/or systematic treatment depending on MF status. The demographic characteristics of patients and healthy individuals and the clinical features of patients are listed in Table 1.

To study the association between rs2910164 (C>G) and rs767649 (T>A) polymorphisms with the expression levels of miR-146a and miR-155, respectively, genotyping was performed in 33/41 MF patients and 26/41 healthy individuals of the initial cohort, where corresponding samples were available. Among these patients, 24/33 were male (~73%) and 9 were female (~27%), with median age equal to 60 years. Furthermore, the genotyping cohort included 24 (~73%) early-stage MF patients and 9 patients with advanced-stage MF (~27%).

### 2.2. Plasma Levels of miR-146a and miR-155 Differ Significantly between Patients and Healthy Controls, and among MF Stages

**Figure 1:**
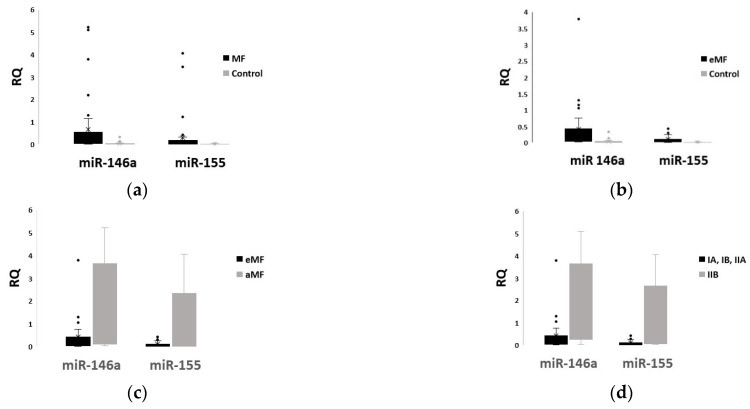
Plasma levels (mean ± SD) of miR-146a and miR-155 in patients vs. control (**a**), in eMF patients vs. control (**b**), in early MF (eMF) vs. advanced MF (aMF) patients (**c**) and in clinical stages IA, IB, IIA vs. IIB (**d**).

The relative quantity (RQ) of both miR-146a and miR-155 was significantly increased in the plasma of MF patients when compared to healthy individuals (*p* = 0.001 and *p* = 0.028, respectively; Figure 1a) and in the plasma of early-stage MF patients vs. healthy controls (*p* = 0.001 for miR-146a and *p* < 0.001 for miR-155; Figure 1b). Moreover, advanced-stage MF showed markedly higher levels of miR-146a and miR-155 in comparison with early-stage MF (*p* = 0.009 and *p* = 0.002, respectively; Figure 1c). Since stage IIB is considered the threshold stage for advanced-stage MF, we also compared plasma levels of miR-146a and miR-155 between patients at clinical stages IA, IB, IIA (comprising early-stage MF) and stage IIB. Interestingly, both miRNAs were significantly upregulated in stage IIB (*p* = 0.008 for miR-146a and *p* = 0.001 for miR-155; Figure 1d). Table S1 summarizes a statistical analysis of miRs expression between groups.

Although analysis with ANOVA revealed that both miRNAs’ levels were significantly different between clinical stages (Figure S1 and Tables S2 and S3 (see Supplementary Materials)), the small sample size in some stages did not allow for post hoc analysis.

**Figure 2:**
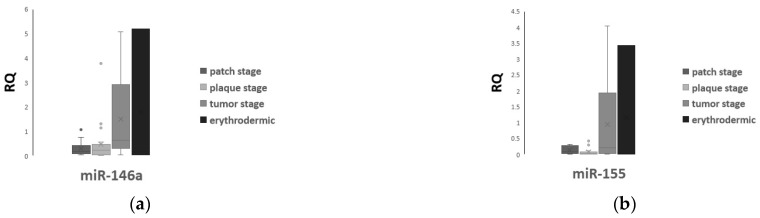
Plasma levels (mean ± SD) of miR-146a (**a**) and miR-155 (**b**) among MF patients with different skin lesions.

To further investigate the association of miR-146a and miR-155 with skin lesions of MF, we compared plasma levels of both miRNAs between patient groups with different manifestations in the skin. Plasma levels of miR-146a seemed to slightly differ (*p* = 0.076) among groups with different clinical skin lesions (Figure 2a and Table S4) but the difference did not reach statistical significance. However, miR-155 displayed markedly different levels among patients with different lesions (*p* = 0.024) (Figure 2b and Table S4). LSD post hoc analysis showed that miR-155 was upregulated in tumor-stage MF when compared with plaque-stage (*p* = 0.018) or patch-stage MF (*p* = 0.039), as well as in erythrodermic patients when compared with plaque-stage (*p* = 0.027) or patch-stage patients (*p* = 0.044, Table S5). No significant difference was found in miR-155 levels between erythrodermic and tumor-stage MF patients, as well as between plaque- and patch-stage MF patients (*p* = 0.701 and *p* = 0.865, respectively, Table S5).

There was no statistically significant difference in the plasma levels of either miRNA between patients who received treatment and patients with no therapy at all (*p* = 0.614 for miR-146a and *p* = 0.243 for miR-155), or between patients receiving different treatment modalities (skin-directed, systemic, or combination therapy) (*p* = 0.446 for miR-146a and *p* = 0.648 for miR-155) (Figure S2). Similarly, sex (*p* = 0.437 for miR-146a and *p* = 0.343 for miR-155) and age (*p* = 0.079 for miR-146a and *p* = 0.336 for miR-155) of patients had no significant influence on miRNA levels.

**Figure 3:**
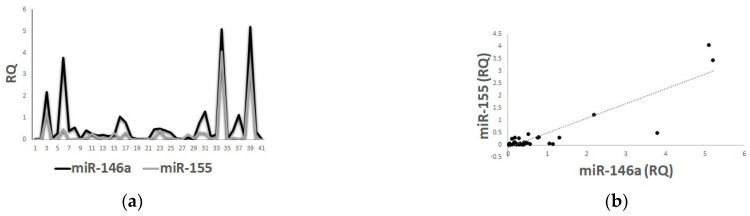
Positive correlation between plasma levels of miR-146a and miR-155 in the patients’ cohort, scatter plot (**a**) and line of tension (**b**).

Interestingly, a notable positive correlation between plasma levels of mir-146a and miR-155 was observed among patients (*p* < 0.001) (Figure 3a,b).

### 2.3. Allelic and Genotypic Patterns of rs2910164 (C>G) and rs767649 (T>A) Polymorphisms Present a Statistically Significant Difference in the Distribution between MF Patients and Healthy Individuals, Displaying a Strong Association with Susceptibility to MF

The allelic and genotypic distribution of miR-146a rs2910164 and miR-155 rs767649 polymorphisms among MF patients and healthy individuals is presented in Table 2. Alignment of the DNA sequence coding for pre-mir-155 did not detect any SNPs in any of the tested samples.

In the genotypic model, the CC genotype of rs2910164 was significantly more frequent in healthy individuals and was associated with a reduced risk of MF (OR = 0.011, 95% CI 0.001–0.107, *p* < 0.01). There was no difference in the distribution of GC genotype between the two study groups (*p* = 0.066). In the dominant model, mutant allele-associated genotypes GC+CC displayed an increased prevalence among healthy individuals and were correlated with a decreased risk of MF (OR = 0.079, 95% CI 0.022–0.290, *p* < 0.01). Furthermore, in the recessive and allelic models, both the CC genotype and C allele showed higher distribution in healthy controls when compared with MF cases and were associated with lower risk of MF (OR = 0.020, 95% CI 0.002–0.166, *p* < 0.01 and OR = 0.074, 95% CI 0.030–0.180, *p* < 0.01).

Regarding rs767649, in the genotypic model, the AA genotype demonstrated higher frequency among MF patients and was associated with increased susceptibility for MF (OR = 8, 95% CI 1.588–40.299, *p* < 0.01). The TA genotype did not display a significant difference in the distribution between the two study groups (OR = 4, 95% CI 0.384–41.701, *p*= 0.217). In the dominant model, an increased prevalence of mutant allele-associated genotypes TA+AA was detected among MF cases and was associated with a high risk of MF (OR = 6.667, 95% CI 1.674–26.554, *p* < 0.01). Moreover, in the recessive and allelic models, the frequencies of the AA genotype and A allele were significantly higher in MF patients and were correlated with an increased risk of MF (OR = 6.857, 95% CI 1.374–34.217, *p* = 0.01 and OR = 4.097, 95% CI 1.386–12.111, *p* < 0.01).

When comparing patients with eMF and aMF (Table S6), there was no significant difference in the distribution of any genotypic and allelic pattern of rs2910164 among these groups. Similarly, for rs767649, there was no difference between aMF and eMF patients in the genotypic, dominant and recessive model. However, in the allelic model, the A allele demonstrated higher distribution among aMF patients and was correlated with increased susceptibility for aMF (OR = 3.143, 95% CI 1.024–9.648, *p* = 0.041).

### 2.4. rs2910164 (C>G) and rs767649 (T>A) Are Associated with Plasma Levels of miR-146a and miR-155, Respectively, and Their Concomitant Homozygous Presence Is Associated with Susceptibility to MF

Assessment of the possible impact of rs2910164 on the plasma levels of miR-146a showed that the transition of genotypes GG→CG→CC was associated with decreased expression of miR-146a (*p* < 0.01 and *r* = −0.551) Regarding the rs767649 polymorphism, the transition of genotypes ΤΤ→AΤ→AA was associated with increased levels of miR-155 (*p* = 0.028 and *r* = 0.286) (Figure S3).

Considering the positive correlation between mir-146a and miR-155 in patients’ plasma and the impact of the SNPs in their expression, we evaluated the distribution of genotypic combinations GG+AA and CC+TT among MF patients and controls (Table 3). The genotypic combination GG+AA was only detected in patients and was correlated with increased susceptibility to MF (OR = 1.49, 95% CI 1.177–1.908, *p* < 0.01). On the contrary, CC+TT genotypic combination presents a higher frequency in healthy individuals and is associated with low risk for MF (OR = 0.025, 95% CI 0.003–0.210, *p* < 0.01).

### 2.5. rs2910164 and rs767649 Could Affect the Secondary Structure and Minimum Free Energy of miR-146a and Regulatory Region of miR-155, Respectively

**Figure 4:**
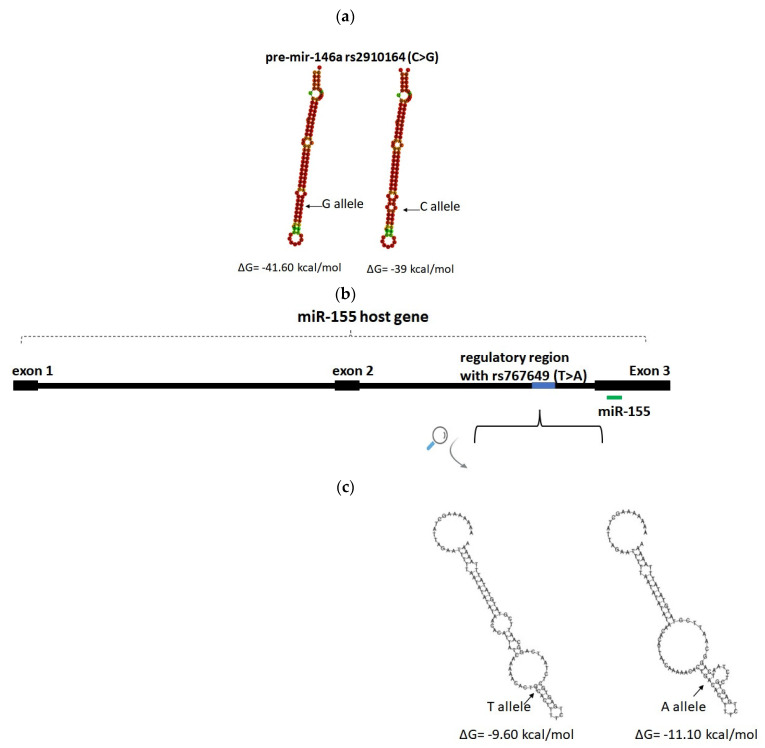
The impact of rs2910164 (C>G) in the secondary structure and minimum free energy of pre-miR-146a, as predicted by RNAfold Web Server (**a**). Genomic location of rs767649 (T>A) in a regulatory region of miR-155 (**b**) and impact of the polymorphism in the secondary structure of single-stranded DNA, as predicted by VectorBuilder (**c**).

No G-quadruplexes or R-loop structures can be formed in the presence of any allele of rs2910164 and rs767649. The mutant allele C of rs2910164 causes a mispairing in the position +60 within the hairpin of pre-mir-146a, increasing the minimum free energy from −41.60 kcal/mol to −39 kcal/mol when compared with the wild-type allele G (Figure 4a). In rs767649, the mutant A allele affects the secondary structure of a regulatory region of miR-155 (Figure 4b), reducing the minimum free energy from −9.60 kcal/mol to −11.10 kcal/mol in comparison with the wild-type allele G (Figure 4c).

Computational analysis of transcription factors (TF) binding prediction revealed TFs that bind in the region around rs767649, in the presence of either the T or A allele (e.g., MEIS1 that presents the highest relative affinity score for both alleles). Other TFs are predicted to bind only in the presence of T allele (Pax2, MEIS3, MEIS2 and MAFG:NFE2L1). Interestingly, T box factors TBX4, TBX3, TBX6, TBX5, TBX1 and TF MGA seem to “recognize” and bind to miR-155 regulatory region only in the presence of the A allele (Table S7).

## 3. Discussion

Histopathological examination of skin lesions, immunohistochemical study, and clinicopathological correlation remain the method of choice for diagnosis of MF [22,23]. Despite the advances in diagnostic markers, the polymorphic nature of MF lesions delays definitive diagnosis and has a negative impact on the disease course [24]. Thus, novel, reliable biomarkers are needed to support a timely diagnosis. Among the many proposed biomolecules, miRNAs have been evaluated as diagnostic and prognostic biomarkers for MF, and some have been identified with a possible role in disease pathogenesis [25].

A panel consisting of miRNAs was originally proposed for the diagnosis of MF by Ralfkiaer et al. in 2011 [26], followed by many studies [16,27,28,29,30,31] recognizing their potential role as biomarkers. Nevertheless, the expression of miRNAs was mainly studied in skin biopsies [9,25,27,32,33,34]. To date, miRNAs plasma levels were only evaluated in two studies [16,17]. In this study, we evaluated the plasma levels of miR-146a and miR-155 in patients with MF and healthy volunteers. We additionally examined how the presence of two SNPs, rs2910164 and rs767649, may affect the levels of miR-146a and miR-155, respectively. Although these SNPs have been associated with many diseases, there was no such correlation study in MF.

The increased plasma levels of miR-146a and miR-155 in MF patients, as compared to healthy individuals, follow a similar expression pattern to skin biopsies [13,14,26,35]. Moreover, our results confirm, in a larger sample, the findings of two previous studies [16,17] detecting increased plasma levels of miR-155 in MF patients. Both miR-146a and miR-155 were found to be significantly upregulated even in early-stage patients, compared to healthy volunteers, rendering them as promising biomarkers for assisting in diagnosis in early stages. Both miRNAs were significantly higher in the plasma of advanced-stage patients when compared to those in early stages. This may be attributed to the increased tumor burden in advanced stages, or to the pathogenetic role of these molecules in disease progression, as, in two studies using cell lines, the activity of STAT5/BIC/miR-155 [13] and JAK/STAT3/STAT4/miR-146a [36] pathways was associated with disease progression. This is further emphasized by the finding that plasma levels of both miRNAs were increased in patients at clinical stage IIB, when compared to patients at lower clinical stages. Similarly, when examining miRNA plasma levels in relation to skin manifestations, patients in erythrodermic and tumor stage had significantly higher miR-155 plasma levels when compared to patients in patch and plaque stages. In a previous study, miR-155 plasma levels were found to be significantly increased in tumor/plaque-stage patients vs. patch-stage patients [16]. To date, miR-146a plasma levels have not been evaluated in MF.

Interestingly, a positive correlation was identified between the plasma levels of the two miRNAs. This could also reflect the tumor burden of each patient or may be the result of a common regulation pathway for the expression of the two miRs. Evidence of a common regulation mechanism is provided by studies in macrophages, where, during the initial stage of activation, NFkBp65 binds in both miR-146a and miR-155 gene loci, increasing their expression, in combination with histone H3 methylation in both genes [37]. Another study suggests that miR-155 and miR-146a form a combined positive and negative regulatory loop controlling NF-κB activity: NF-κB, activated by inflammatory stimuli, rapidly increases miR-155 expression. miR-155 then acts as an amplifier and positive regulator to ensure robust NF-κB activity. miR-146a levels rise more slowly and negatively regulate NF-κB (by targeting IRAK and TRAF6), resulting in a decrease in miR-155 levels [38]. Nevertheless, further clarification of such mechanisms by bioinformatic and functional studies in cutaneous lymphoma cell lines is required. 

The single-nucleotide polymorphism rs2910164 (C>G) is located at the +60 nucleotide position of pre-mir-146a, corresponding to the seed area of mature miR-146a. According to the NCBI SNP database, the G allele is more frequent than C, which is also confirmed by our study. Interestingly, the C allele and CC genotype are significantly more frequent among healthy volunteers than among MF patients and are associated with reduced MF risk. Moreover, the C allele and CC genotype are associated with reduced miR-146a plasma levels, in line with previous studies [18,39,40,41,42]. This reduction in miR-146a levels in the presence of C allele could be attributed to the altered pri- and/or pre-miR-146a processing [18] associated with an altered secondary conformation and free energy. This conformation contains an additional loop and may affect the protein binding and stability of the molecule. Moreover, the presence of the SNP in the miR’s seed area may affect its binding with mRNA, altering its target genes and pathways and contributing to the disease pathogenesis.

Single-nucleotide polymorphism rs767649 (T>A) is located in a regulatory region for miR-155. The dominant allele is T, and it is also detected with a higher frequency in our samples. In the study cohort, the A allele and AA genotype were more frequent among MF patients and were associated with increased MF risk and with increased miR-155 plasma levels. Although in many studies, the A allele is associated with increased miR-155 expression [20,43,44], the results are controversial as there are also studies associating the A allele with reduced expression [21,45]. The position of the SNP (1570 nucleotides upstream of miR-155) is recognized as a regulatory region for miR-155, localized to the second intron of *BIC* (host gene). In the presence of the A allele, there is an alteration in the conformation of the single-stranded DNA around the polymorphism and its free energy. Alternative secondary structures of single-stranded DNA, formed by DNA unwinding during transcription, can affect the binding of RNA polymerase and other enzymes, and may cause RNA polymerase to pause or stall, affecting transcription. Stable structures are more prone to promoter-proximal pausing [46]. Alternatively, the presence of the A allele could alter the binding of transcription factors. The T-Box family of transcription factors, which participate in development and cancer (including lymphomas [47]), are predicted to bind only in the presence of the A allele. These findings of in silico analysis are only suggestive of plausible mechanisms that could explain our results, and should, therefore, be confirmed experimentally.

Finally, the positive correlation between miRNAs levels and the correlation between genotypes and expression suggest a possible synergistic role of the two SNPs as predisposing factors for MF. This cannot be overlooked, since the genotypic combination GG + AA is only detected in patients and is associated with increased disease risk. Combined genotypes of SNPs in separate genes can act synergistically or antagonistically and constitute overlapping risk factors for benign and malignant diseases [48,49].

## 4. Materials and Methods

### 4.1. Clinical Samples

Peripheral blood from 41 MF patients and 41 healthy individuals was collected in EDTA. Both newly diagnosed and pre-treated patients with active disease were included in the study cohort. Patients were staged according to WHO-EORTC classification [50] and had no history of autoimmune disease, other malignancies, or recent infection. Healthy volunteers had no history of autoimmune disease, other malignancies, or recent infection and their demographic characteristics were similar to the patient cohort. All patients were followed in the Cutaneous Lymphoma Clinic at “Papageorgiou” Hospital, Thessaloniki, Greece.

Blood samples were centrifuged at 2000× *g* at 4 °C for 15 min for plasma separation. For genotyping, DNA was extracted from white blood cells of 33/41 MF patients and 26/41 controls.

### 4.2. microRNAs Isolation

miRNAs were isolated from 200 μL plasma using miRNeasy Serum/Plasma Kit with QIAzol Lysis reagent (Qiagen, Hilden, Germany) according to the protocol. During purification, cel-miR-39 (Caenorhabditis elegans miR-39) was added as spike-in control. The quantity and quality (purity) of the isolated total RNA was assessed spectrophotometrically, with measurements at wavelengths of 230, 260, and 280 nm.

### 4.3. Reverse Transcription and Quantitative Real-Time Polymerase Chain Reaction

Reverse transcription (RT) and quantitative real-time PCR (qPCR) were carried out using TaqMan miRNA assays (assay ID: 0468 for hsa-miR-146a and 475062_mat for hsa-miR-155, Applied Biosystems, Waltham, MA, USA) using TaqMan microRNA Reverse Transcription kit, and TaqMan Universal PCR master mix with UNG (Applied Biosystems). Amplification was carried out in a StepOnePlus Real Time PCR system (Applied Biosystems). The cycling conditions were 2 min at 50 °C, 10 min at 95 °C, followed by 40 cycles of 15 s at 95 °C and 60 s at 60 °C. All samples were run in duplicate. The relative quantity (RQ) of miRNAs in the plasma was calculated using the 2^–ΔCT^ [2^–(CTgene of interest– CTreference gene)^] method, using cel-miR-39 as exogenous control.

### 4.4. Genotyping

Genomic DNA was isolated from 100 μL peripheral blood using Monarch Genomic DNA Purification Kit (New England Biolabs, Ipswich, MA, USA). Sequences containing the SNPs were amplified by PCR using KAPA HiFi (Kapa Biosystems, Cape Town, South Africa), followed by Sanger sequencing. Cycling conditions included an initial denaturation at 95 °C for 3 min, followed by 25 cycles of denaturation at 98 °C for 20 s, annealing at 58 °C for 15 s (rs2910164, pre-mir-155) or at 62 °C for 13 s (rs767649), and extension at 72 °C for 15 s. The PCR primers and conditions used in the study are summarized in Table 4.

### 4.5. Sample Size Calculation

The optimal sample size required to detect a statistically significant difference in the plasma levels of miR-146a and miR-155 was determined by a pilot study, in which an effect size of 0.78 was added to a statistical power of 94% (1-*b* = 0.94) and a level of significance equal to a = 0.05. Data were analyzed with two tailed Student’s *t*-test using G*Power 3.1.9.2. for Windows software [51]. The optimal sample size was calculated at *n* = 82, with a ratio of MF patients/healthy individuals equal to 1:1, i.e., 41 patients and 41 controls.

### 4.6. Statistical Analysis

RQ values were used to evaluate differences between miRNAs’ expression. Normal distribution was examined using the Shapiro–Wilk test and Kolmogorov–Smirnov test. Both tests provided evidence in favor of normal distribution (*p* > 0.05). Homogeneity of variance was examined using Levene’s test, which supports equality of variances (*p* > 0.05). A two-tailed Student’s *t*-test or one way analysis of variance, followed by Fisher’s Least Significant Difference post hoc test, was used to investigate differences in mean expression levels. Associations between clinical characteristics and miRNAs’ levels were assessed by Pearson correlation analysis. The genotype and allele frequency differences among study groups were examined using x^2^ test. Associations between Mycosis Fungoides risk and genotype/allele patterns were estimated by odds ratios (ORs) and 95% confidence intervals (CIs). The Spearman correlation coefficient was used to evaluate correlations between miRNAs’ levels with the presence of polymorphisms. Different genetic models were recruited to examine the allelic/genotypic distribution between MF patients and healthy controls and the association with MF risk. All four models that were used are described below:

(I) The genotypic model, in which the wild-type homozygous genotype was used as a reference (GG for rs2910164 and TT for rs767649) to investigate distribution when compared with the other two genotypes (heterozygous genotype GC for rs2910164 and AT for rs767649, and mutant homozygous genotype CC for rs2910164 and AA for rs767649) in patients and healthy controls.
(II) The dominant model, in which a wild-type homozygous genotype was used as a reference (GG for rs2910164 and TT for rs767649) to investigate the prevalence of mutant-allele-associated genotypes (CC+CG for rs2910164 and AA+AT for rs767649) within MF cases and controls.
(III) The recessive model, in which wild-type-allele-associated genotypes (GC+GG for rs2910164 and AT+TT for rs767649) were used as a reference to investigate the prevalence of the mutant homozygous genotype (CC for rs2910164 and AA for rs767649) within MF and control groups.
(IV) The allelic model, in which the wild-type allele (G for rs2910164 and T for rs767649) was used as a reference to investigate the prevalence of the mutant allele (C for rs2910164 and A for rs767649) within MF and control groups.

IBM SPPS version 25 software was used, and all tests were provided under a 5% level of significance.

### 4.7. Bioinformatic Analysis

The sequence of pre-mir-146a containing the rs2910164 polymorphism and in the regulatory region of miR-155 containing the rs767649 polymorphism was examined. The prediction of G-quadruplexes (G4) in pre-mir-146a sequence and in the regulatory region of miR-155 polymorphism was performed with G4Hunter online tool [52].

The web tools QmRLFS-finder [53] and Emboss Palindrome [54] were used for the detection of the R-loop-forming structure and inverted repeats (stem loops), respectively.

The online tools RNAfold Web Server [55] and VectorBuilder were utilized to predict the effect of rs2910164 and rs767649 polymorphisms in the secondary structure and minimum free energy (MFE) of pre-mir-146a and the regulatory region of miR-155, respectively.

Jaspar Core database [56] was used to predict, in silico, the impact of rs767649 (T>A) in the binding affinity of transcription factors, setting the following sequence as the input: ACACTG(T/A)CACTTT. The relative score threshold was set at 85%.

## 5. Conclusions

The evaluation of mir-146a and mir-155 plasma levels in MF patients seems to be of diagnostic and prognostic value and is emerging as a putative, noninvasive biomarker. Moreover, the presence of SNPs is closely associated with miR expression and possibly disease susceptibility, as revealed by the present study. Unveiling the mechanism by which SNPs can affect miR’s expression and function may be an important step towards further understanding the pathogenesis of MF and other diseases.

## Figures and Tables

**Table 1:** Demographic and clinical characteristics of the patients and healthy individuals included in the study.

	Patients	Healthy Individuals
	*n* = 41	*n* = 41
**Demographic characteristics**		
Median age (range)	59 (39–85)	56 (34–79)
Male *n* (%)	30 (73.17%)	28 (68.29%)
Female *n* (%)	11 (26.83%)	13 (31.71%)
**Clinical Features**	***n* (%)**	
Patch stage	11 (27%)	
Plaque stage	21 (51%)	
Tumor Stage	6 (14%)	
Erythrodermic	3 (8%)	
**MF stage**	***n* (%)**	
Early-stage (IA–IIA)	32 (78.03%)	
ΙA	22 (53.65%)	
ΙΒ	8 (19.51%)	
ΙΙA	2 (4.87%)	
Advanced-stage (IIB–IV)	9 (21.97%)	
ΙΙΒ	5 (12.19%)	
ΙΙΙ	3 (7.31%)	
ΙV	1 (2.47%)	
**Treatment**	***n* (%)**	
None	19 (46.3%)	
Skin-directed	3 (7.3%)	
Systematic	12 (29.4%)	
Combination of skin-directed and systematic	7 (17%)	

**Table 2:** Allelic and genotypic distribution of miR-146a rs2910164 (C>G) and miR-155 rs767649 (T>A) between MF patients and controls.

**miR-146a rs2910164 (C>G)**			
**Genotypes and Alleles**	**MF Cases** ***n* = 33 (%)**	**Controls** ***n* = 26 (%)**	***p*** **-Value, Unadjusted OR (95% CI)**
GG	23 (69.7)	4 (15.3)	1 (reference)
GC	9 (27.3)	6 (23)	*p* = 0.066, 0.261 (0.059–1.148)
CC	1 (3)	16 (61.7)	*p* < 0.01, 0.011 (0.001–0.107)
Dominant model			
GG	23 (69.7)	4 (15.3)	1 (reference)
GC+CC	10 (30.3)	22 (84.7)	*p* < 0.01, 0.079 (0.022–0.290)
Recessive model			
GC+GG	32 (97)	10 (38.3)	1 (reference)
CC	1 (3)	16 (61.7)	*p* < 0.01, 0.020 (0.002–0.166)
Allelic model			
G	55 (83.4)	14 (26.9)	1 (reference)
C	11 (16.6)	38 (73.1)	*p* < 0.01, 0.074 (0.030–0.180)
**miR-155 rs767649 (T>A)**			
**Genotypes and Alleles**	**MF Cases** ***n* = 33 (%)**	**Controls** ***n* = 26 (%)**	***p*** **-Value, Unadjusted OR (95% CI)**
TT	18 (54.5)	23 (88.3)	1 (reference)
TA	3 (9)	1 (4)	*p* = 0.217, 4 (0.384–41.701)
AA	12 (36.5)	2 (7.7)	*p* < 0.01, 8 (1.588–40.299)
Dominant model			
TT	18 (54.5)	23 (88.3)	1 (reference)
ΤA+AA	15 (45.5)	3 (11.7)	*p* < 0.01, 6.667 (1.674–26.554)
Recessive model			
ΤA+TT	21 (63.5)	24 (92.3)	1 (reference)
AA	12 (36.5)	2 (7.7)	*p* = 0.01, 6.857 (1.374–34.217)
Allelic model			
T	39 (59)	47 (90.4)	1 (reference)
A	27 (41)	5 (9.6)	*p* < 0.01, 6.679 (2.347–19.003)

**Table 3:** Distribution of genotypic combinations of rs2910164 and rs767649 in MF patients and controls.

Genotypic Combinations	MF *n* = 33 (%)	Controls *n* = 26 (%)	*p*-Value, Unadjusted OR (95% CI)
Rest cases	22 (67)	26 (0)	1 (reference)
GG+AA	11 (33)	0 (0)	*p* < 0.01, 1.49 (1.177–1.908)
Rest cases	32 (97)	12 (46)	1 (reference)
CC+TT	1 (3)	14 (54)	*p* < 0.01, 0.025 (0.003–0.210)

**Table 4:** Primers and conditions for PCR, size and genomic location of the products.

Target	Primer Sequence 5′→3′	Product Length	Genomic Location	Annealing Temperature
rs2910164	F: CATTGGATTTACCAGGCTTTT R: CACACTCCTTATACCTTCAGAGC	305 bp	Chr5, GRCh38.p14 (160485260, 160485564)	58 °C
pre-mir-155	F: GCATACACAAACATTTCTTTCTCTCT R: CATCCCAGTGACCAGATTATGA	329 bp	Chr21, GRCh38.p14 (25573862, 25574191)	58 °C
rs767649	F: ACAAAAGGGGACCTGTGTGA R: TTGAAGGTAAATTGCTGGCATACT	281 bp	Chr21, GRCh38.p14 (25572299, 25572579)	62 °C

## Data Availability

All data are available from the corresponding author upon reasonable request.

## Supplementary Materials

The following supporting information can be downloaded at: https://www.mdpi.com/article/10.3390/ijms24010271/s1.
