# Supplementary material for: Dysregulation of Plasma miR-146a and miR-155 Expression Profile in Mycosis Fungoides Is Associated with rs2910164 and rs767649 Polymorphisms
Source: Int J Mol Sci. 2022 Dec 23;24(1):271. doi: 10.3390/ijms24010271 (PMC9820385; doi:10.3390/ijms24010271)
Supplement: Supplementary file 1 [file ijms-24-00271-s001.zip › ijms-2078148-supplementary.pdf]

# Supplementary Materials

**Table S1.** Descriptive statistics and statistical significance for equality of expression levels (relative quantity, RQ) of miR-146a and miR-155 in plasma samples of MF patients vs healthy controls(a), of early MF patients vs controls (b), of early vs advanced MF patients (c) and of stages IA, IB, IIA vs stage IIB patients (d).

| Compared groups | | n | Mean RQ | SD | *p*-value | Mean RQ | SD | *p*-value |
|---|---|---|---|---|---|---|---|---|
| | | | | **Mir-146a** | | | **Mir-155** | |
| a | Patients | 41 | 0.674212 | 1.2356 | 0.001 | 0.29918 | 0.82278 | 0.028 |
| | Controls | 41 | 0.035288 | 0.0624 | | 0.01112 | 0.01362 | |
| b | eMF patients | 32 | 0.414570 | 0.70222 | 0.001 | 0.09590 | 0.13090 | <0.001 |
| | Controls | 41 | 0.035288 | 0.06244 | | 0.01112 | 0.01362 | |
| c | eMF patients | 32 | 0.414570 | 0.70222 | 0.009 | 0.09590 | 0.13090 | 0.002 |
| | aMF patients | 9 | 1.59738 | 2.12008 | | 1.0219 | 1.6017 | |
| d | Stages IA, IB, IIA | 32 | 0.41457 | 0.70222 | 0.008 | 0.10328 | 0.13406 | 0.001 |
| | Stage IIB | 5 | 1.7188 | 2.051 | | 1.1428 | 1.699 | |

SD: Standard deviation, eMF: early MF (stages IA-IIA), aMF: advanced MF (stages IIB-IV).

**Table S2.** Descriptive statistics and statistical significance for equality regarding plasma expression levels (relative quantity; RQ) of miR-146a and miR-155 among patients at different clinical stages of disease.

| | | Sum of squares (RQ values) | Df | Mean square (RQ values) | F ratio | *p*-value |
|---|---|---|---|---|---|---|
| **MiR-146a** | Between Groups | 20.528 | 5 | 4.106 | 3.544 | 0.011 |
| | Within Groups | 40.544 | 35 | 1.158 | | |
| | Total | 61.072 | 40 | | | |
| **MiR-155** | Between Groups | 7.155 | 5 | 1,431 | 2.523 | 0.047 |
| | Within Groups | 19.854 | 35 | 0.567 | | |
| | Total | 27.009 | 40 | | | |

Df: Degrees of freedom.

**Table S3.** Mean values regarding expression levels (relative quantity; RQ) of miR-146a and miR-155 in plasma samples of MF patients at different clinical stages of disease.

| Clinical stage of MF | Number of MF patients | Mean RQ value (miR-146a) | Mean RQ value (miR-155) |
|---|---|---|---|
| IA | 22 | 0.24858653 | 0.079568744 |
| IB | 8 | 0.358127215 | 0.130371441 |
| IIA | 2 | 0.96617444 | 0.255880175 |
| IIB | 5 | 2.318894054 | 1.14283346 |
| III | 3 | 1.797237239 | 1.159176468 |
| IV | 1 | 0.390245 | 0.005940587 |

**Table S4.** Descriptive statistics and statistical significance for equality regarding plasma expression levels (relative quantity; RQ) of miR-146a and miR-155 among patients with different clinical features.

| | | Sum of squares (RQ values) | Df | Mean square (RQ values) | F ratio | *p*-value |
|---|---|---|---|---|---|---|
| **MiR-146a** | Between Groups | 10.237 | 3 | 3.412 | 2.484 | 0.076 |
| | Within Groups | 50.835 | 37 | 1.374 | | |
| | Total | 61.072 | 40 | | | |
| **MiR-155** | Between Groups | 6.030 | 3 | 2.010 | 3.545 | 0.024 |
| | Within Groups | 20.979 | 37 | 0.567 | | |
| | Total | 27.009 | 40 | | | |

Df: Degrees of freedom.

**Table S5.** LSD post hoc analysis and statistical significance regarding plasma expression levels (relative quantity; RQ) of miR-155 among patients with different clinical features.

| Multiple comparisons (clinical stages) | | *p*-value |
|---|---|---|
| Plaque stage | Tumor stage | 0.018 |
| | Patch stage | 0.865 |
| | Erythrodermic | 0.027 |
| Tumor stage | Plaque stage | 0.018 |
| | Patch stage | 0.039 |
| | Erythrodermic | 0.701 |
| Patch stage | Plaque stage | 0.865 |
| | Tumor stage | 0,039 |
| | Erythrodermic | 0,044 |
| Erythrodermic | Plaque stage | 0,027 |
| | Tumor stage | 0,701 |
| | Patch stage | 0,044 |

**Table S6.** Genotype and allele distribution of miR-146a rs2910164 (C>G) and miR-155 rs767649 (T>A) between aMF and eMF patients.

| miR-146a rs2910164 (C>G) | | | |
|---|---|---|---|
| **Genotypes and alleles** | **aMF cases n=9 (%)** | **eMF cases n=24 (%)** | ***p*-value, unadjusted OR (95% CI)** |
| GG | 6 (66,6) | 17 (70.8) | 1 reference |
| GC | 3 (33.4) | 6 (25) | *p*=0.682, 1.417 (0.267-7.521) |
| CC | 0 (0) | 1 (4.2) | *p*=0.555, 1.059 (0.947-1.184) |
| Dominant model | | | |
| GG | 6 (66,6) | 17 (70.8) | 1 reference |
| GC+CC | 3 (33.4) | 7 (29.2) | *p*=0.817, 1.214 (0.235-6.271) |
| Recessive model | | | |
| GC+GG | 9 (100) | 23 (95.8) | 1 reference |
| CC | 0 (0) | 1 (4.2) | *p*=0.534, 1.043 (0.960-1.134) |
| Allelic model | | | |
| G | 15 (83.4) | 40 (83.4) | 1 reference |
| C | 3 (16.6) | 8 (16.6) | *p*=1, 1 (0.234-4.278) |
| **miR-155 rs767649 (T>A)** | | | |
| **Genotypes and alleles** | **aMF cases n=9 (%)** | **eMF cases n=24 (%)** | ***p*-value, unadjusted OR (95% CI)** |
| TT | 3 (33.3) | 15 (62.5) | 1 reference |
| TA | 1 (11.1) | 2 (8.3) | *p*=0.496, 2.5 (0.168-37.260) |
| AA | 5 (55.6) | 7 (29.2) | *p*=0.129, 3.571 (0.659-19.341) |
| Dominant model | | | |
| TT | 3 (33.3) | 15 (62.5) | 1 reference |
| TA+AA | 6 (66.7) | 9 (37.5) | *p*=0.134, 3.33 (0.664-16.736) |
| Recessive model | | | |
| TA+TT | 4 (44.4) | 17 (70.8) | 1 reference |
| AA | 5 (55.6) | 7 (29.2) | *p*=0.160, 3.036 (0.624-14.771) |
| Allelic Model | | | |
| T | 7 (38.8) | 32 (66.6) | 1 reference |
| A | 11 (61.2) | 16 (33.4) | *p*=0.041, 3.143 (1.024-9.648) |

**Table S7.** Transcription factor binding site profiles for rs767649 polymorphism, as predicted from Jaspar database analysis.

| | | **Target Sequence:** ACACTG**T**CACTTT | | |
|---|---|---|---|---|
| **Matrix ID** | **Name** | **Relative score** | **Strand** | **Predicted Sequence** |
| MA0498.2 | MEIS1 | 0.9713 | - | GTGACAG |
| MA0067.1 | Pax2 | 0.9461 | + | TGTCACTT |
| MA0775.1 | MEIS3 | 0.9449 | - | GTGACAGT |
| MA0442.1 | SOX10 | 0.9068 | - | CAGTGT |
| MA0774.1 | MEIS2 | 0.8881 | - | GTGACAGT |
| MA1536.1 | NR2C2 | 0.8598 | + | ACTGTCAC |
| MA0089.1 | MAFG:NFE2L1 | 0.8557 | - | AGTGAC |
| MA0442.1 | SOX10 | 0.8513 | + | CACTGT |
| | | **Target Sequence:** ACACTG**A**CACTTT | | |
| **Matrix ID** | **Name** | **Relative score** | **Strand** | **Predicted Sequence** |
| MA0498.2 | MEIS1 | 0.9700 | + | CTGACAC |
| MA1536.1 | NR2C2 | 0.9167 | - | AGTGTCAG |
| MA0806.1 | TBX4 | 0.9120 | - | AAGTGTCA |
| MA0442.1 | SOX10 | 0.9068 | - | CAGTGT |
| MA1566.1 | TBX3 | 0.8941 | - | AAAGTGTCAG |
| MA1567.1 | TBX6 | 0.8879 | - | AAAGTGTCAG |
| MA0807.1 | TBX5 | 0.8860 | - | AAGTGTCA |
| MA1535.1 | NR2C1 | 0.8843 | - | AAGTGTCAG |
| MA0801.1 | TBX1 | 0.8786 | - | AAGTGTCA |
| MA0805.1 | MGA | 0.8746 | - | AAGTGTCA |

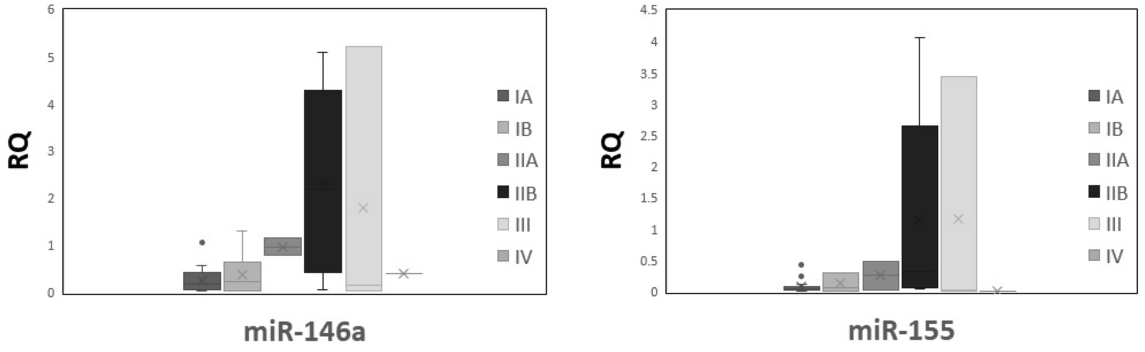


(**a**) (**b**)

**Figure S1.** Plasma expression levels (mean ± SD) of miR-146a (**a**) and miR-155 (**b**) among MF patients at different clinical stages.

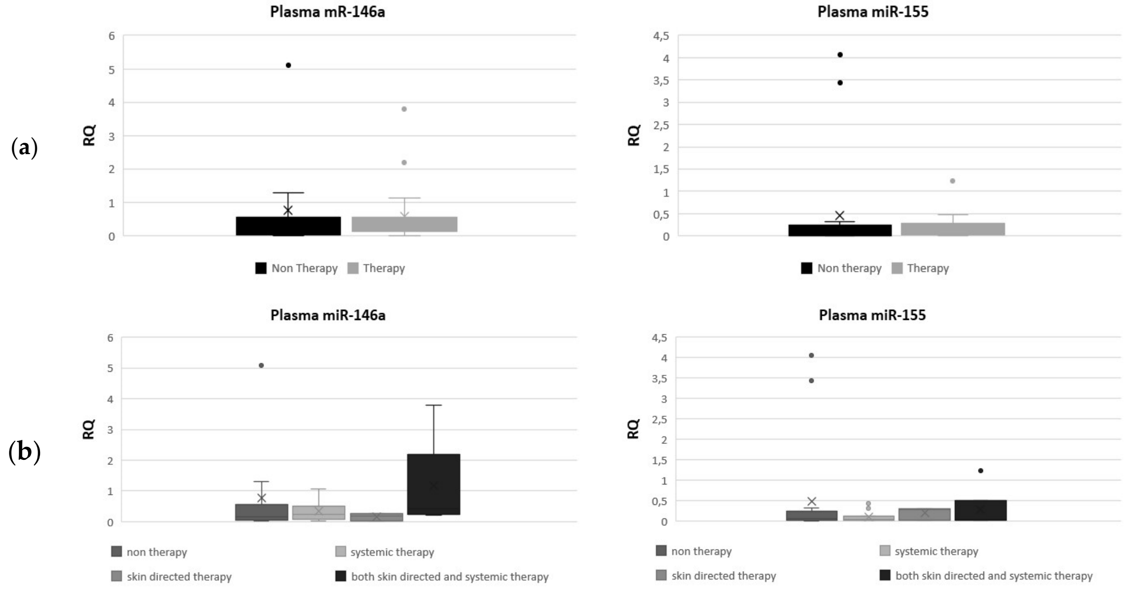


**Figure S2.** Plasma expression levels of miR-146a and miR-155 (mean ± SD) between MF patients with and without therapy (**a**), between MF patients with different treatment regiments (**b**).

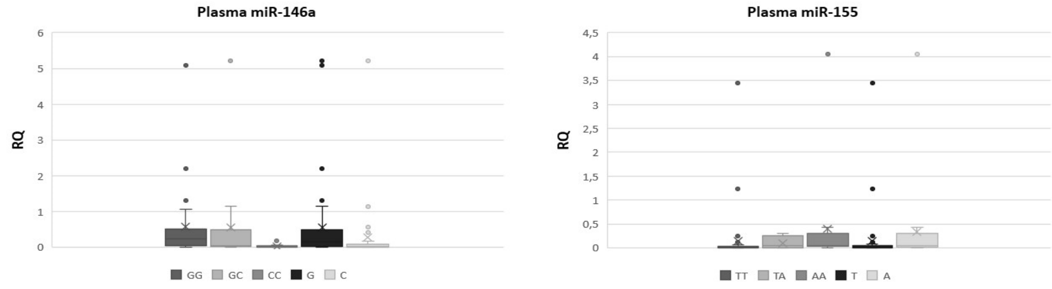


(**a**) (**b**)

**Figure S3.** Association between genotypic/allelic patterns of rs2910164 (C>G) and rs767649 (T>A) polymorphisms withs plasma expression levels of miR-146a (**a**) and miR-155 (**b**) respectively.
